# Maryland Dentists and the Vulcanite Patent

**Published:** 1881-01

**Authors:** 


"Maryland Dentists and the Vulcanite Patent.

A meeting of dentists of the State was held at the Dental College, Lexington and Eutaw streets, last night, in order to inaugurate a combined effort to prevent the extension of the patent of the Goodyear Dental Vulcanite Company. Dr. Henry H. Keech was chosen chairman and Dr. R. B. Winder was appointed secretary. Dr. Winder urged upon the meeting the importance of defeating the proposed extension of the patent, and said that dentists were paying license fees to the Vulcanite Company ranging from $75 to $1,000. He said that it was essential that full statistics bearing upon the subject should be gathered, collated and presented to Congress. Dr. F. J. S. Gorgas, T. S. Waters, E. P. Keech and others discussed the subject. It was stated that the Goodyear patent was thirty years old, but that for ten years past none but dentists have paid any royalty thereon. If the matter was properly presented to Congressmen it was believed the profession would be relieved also. A committee, consisting of Drs. F. J. S. Gorgas, E. P. Keech, S. T. Henkle, C. E. Duck, T. S. Waters and H. H. Keech was appointed to prepare and present a statement of facts in connection with the matter to the representatives of Maryland in the Congress of the United States. The meeting adjourned subject to the call of the chairman."

